# Assessment of the Food Habits of the Moroccan Dorcas Gazelle in M’Sabih Talaa, West Central Morocco, Using the *trn*L Approach

**DOI:** 10.1371/journal.pone.0035643

**Published:** 2012-04-27

**Authors:** Moulay Abdeljalil Ait Baamrane, Wasim Shehzad, Ahmed Ouhammou, Abdelaziz Abbad, Mohamed Naimi, Eric Coissac, Pierre Taberlet, Mohammed Znari

**Affiliations:** 1 Laboratoire Biodiversité et Dynamique des Ecosystèmes [BioDEcos], Faculté des Sciences-Semlalia, Université Cadi Ayyad, Marrakech, Morocco; 2 Laboratoire d’Ecologie Alpine [LECA], CNRS UMR 5553, Université Joseph Fourier, Grenoble, France; 3 Laboratoire Ecologie et Environnement [L2E], Faculté des Sciences-Semlalia, Université Cadi Ayyad, Marrakech, Morocco; 4 Laboratoire de Biotechnologies, Protection et Valorisation des Ressources Végétales [Biotec-VRV], Faculté des Sciences-Semlalia, Université Cadi Ayyad, Marrakech, Morocco; Northwestern University, United States of America

## Abstract

Food habits of the Moroccan dorcas gazelle, *Gazella dorcas massaesyla,* previously investigated in the 1980s using microhistological fecal analysis, in the M’Sabih Talaa Reserve, west central Morocco, were re-evaluated over three seasons (spring, summer and autumn 2009) using the *trn*L approach to determine the diet composition and its seasonal variation from fecal samples. Taxonomic identification was carried out using the identification originating from the database built from EMBL and the list of plant species within the reserve. The total taxonomic richness in the reserve was 130 instead of 171 species in the 1980s. The diet composition revealed to be much more diversified (71 plant taxa belonging to 57 genus and 29 families) than it was 22 years ago (29 identified taxa). Thirty-four taxa were newly identified in the diet while 13 reported in 1986–87 were not found. Moroccan dorcas gazelle showed a high preference to *Acacia gummifera, Anagallis arvensis*, *Glebionis coronaria, Cladanthus arabicus, Diplotaxis tenuisiliqua, Erodium salzmannii, Limonium thouini, Lotus arenarius and Zizyphus lotus*. Seasonal variations occurred in both number (40–41 taxa in spring-summer and 49 taxa in autumn *vs*. respectively 23–22 and 26 in 1986–1987) and taxonomic type of eaten plant taxa. This dietary diversification could be attributed either to the difference in methods of analysis, *trn*L approach having a higher taxonomic resolution, or a potential change in nutritional quality of plants over time.

## Introduction

The knowledge of an endangered species diet is of prime importance to understanding its place in a biological community and improves our comprehension of the functioning of the ecosystem as a whole [1] which is valuable to structuring effective management plans for the conservation of both ecosystem and animal species [2]–[4]. Several methods have been developed to evaluate the composition of herbivore diets: direct observation of animal foraging behavior, and indirect methods of dietary reconstitution (fecal based analysis). The direct observation of animals, when possible, is the more accurate sampling method but is exposed to several potential problems. It could be very difficult to identify accurately the items consumed by an individual when dealing with elusive or nocturnal animals, or when an herbivore feeds in complex environments with many plant species that are not separated spatially [4]. The presence of the observer can also change the behavior of animals [5]. This method is classically time-consuming and the observer can only sample a small number of individuals at a time [5]. To mitigate the problems encountered with direct observations, scientists rely on indirect methods of evaluating diet [6], [7]. Feces based analysis represents the most non-invasive used technique. At present, four fecal-based techniques can be distinguished. The microhistological examination of plant cuticle fragments in pellet samples is the most extensively employed technique [8], [9] in spite of the considerable amount of training required by this method. The second technique is based on natural alkanes of plant cuticular wax and has been used to estimate diet composition of domestic animals [10]–[12] and, less frequently, of wild herbivores [13]–[15]. This technique is limited when the animals feed in complex environment [16]. The Near Infrared Reflectance Spectroscopy (NIRS) is another technique used to predict the composition of herbivores’ diet. However, this method is technically limited by the size and the homogeneity of particles which can bias the analysis [17], [18]. Finally, the DNA based technique for species identification is a relatively new concept [19]–[21]. It provides an alternative mean of studying the diets of wild animals by targeting plant and animal DNA +fragments that are highly variable and that allow taxonomic identification via their “DNA barcode” [4], [22], [23].

Dorcas gazelle has been classified as Vulnerable by the Species Survival Commission of the World Conservation Union [24] since 1988 and considered as endangered in Morocco [25], [26]. The Moroccan dorcas gazelle (*Gazella dorcas massaesyla*) is endemic to the Atlantic plateaux’s [27]. The last surviving wild population of this subspecies is gathered in the M’Sabih Talaa Reserve, north of the Atlas Mountains, where live a herd of about 100 individuals [28]. Some demographic characteristics of this population were investigated [29], [30] and seasonal variation of its food habits was also examined more than 20 years ago (1987) using the microhistological fecal analysis [31]. According to this later study, it seems that the Moroccan dorcas gazelle is rather a specialist herbivore feeding on a small subset of plant species independently of their availability.

Freeland and Jansen [32] and Westoby [33] have shown that large herbivores maximize nutrient intake by selecting a wide range of forage species because of the complementarity in nutrient availability among plant species. Furthermore, Freeland and Jansen [32] have proposed that herbivores avoid exceeding toxic thresholds of secondary plant metabolites by feeding on a variety of plant species. On the basis of crude protein in the feces that were higher than that measured in the plant species, Dorcas gazelle in the Negev desert, Israel, have been suggested to select their diet at the level of plant parts, not species [34]. In addition, and due to its small body size and its feeding mode of intermediate type «Grazer-Browser», the dorcas gazelle would require food of a relatively high nutritional quality to satisfy its needs which would affect the food items selected.

In this paper, we present the first use of the increasingly popular non-invasive genetic technique, the *trn*L approach [35]–[40] to determine composition of the diet and its seasonal variation in the Moroccan dorcas gazelle in the M’Sabih Talaa Reserve. The obtained results are compared to those previously reported by Loggers [31] for the same population 22 years ago using the microhistological method. The obtained results will be taken into account in elaborating a conservation and management plan of the studied population and its habitat.

## Materials and Methods

### Ethics Statement

There is no need for an ethics statement, as our present research work did not involve capture or any direct manipulation or disturbance of animals. We only collected samples of plants and feces for molecular analyses. The access to the reserve was under permission of the *Haut Commissariat aux Eaux et Forêts et à la Lutte Contre la Désertification* [*HCEFLCD*] that is responsible for the management of protected areas and wildlife in Morocco. For our fieldwork we were not allowed to capture or to disturb gazelles.

### Study Area

**Figure 1:**
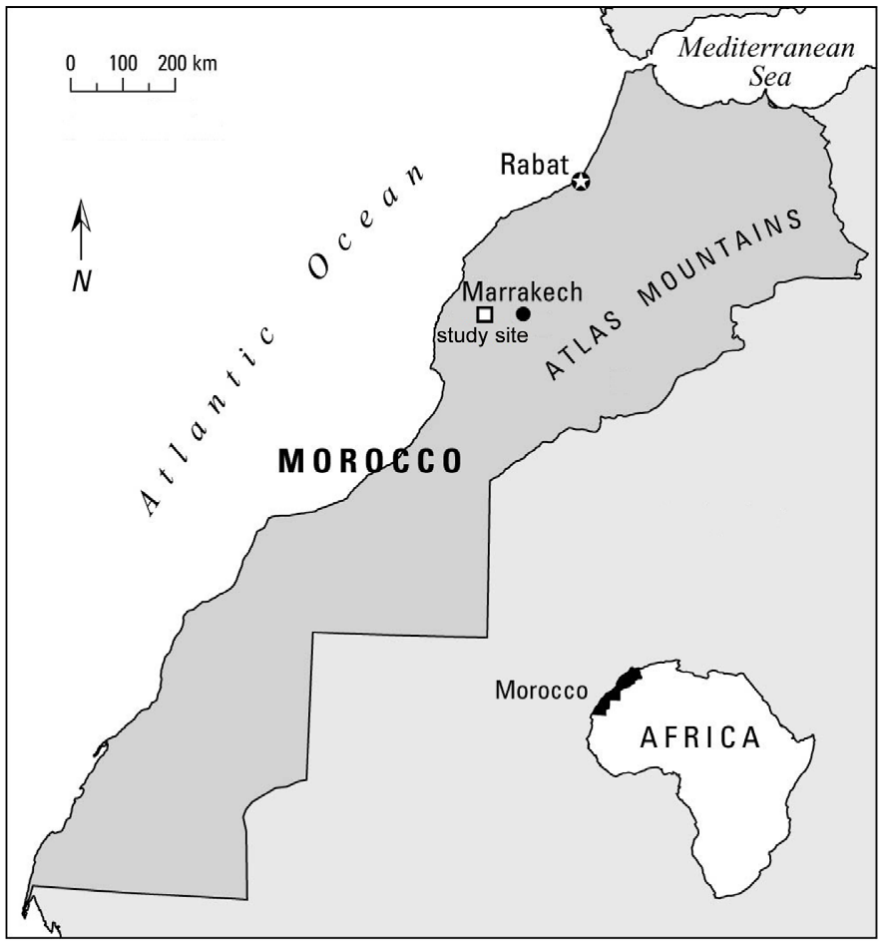
Map showing the geographic location of the M’Sabih Talaa Reserve, West central Morocco. The location of the study site is indicated by the square.

The study was conducted in the M’Sabih Talaa (MT) Reserve, North West of the Atlas Mountains, Morocco (Fig. 1). The MT Reserve is a part of the Haouz arid plain (31°48′N- 8°30′ W, 380 m a.s.l.), it is situated 68 km west of Marrakech and 80 km south east of Safi. The habitat is dominated by *Stipa retorta* grasslands dotted with *Zizyphus lotus* and *Retama monosperma* shrubs [31]. This reserve was created to be a zone of experiment with the aim of testing to what extent some plants (trees, shrubs and herbaceous plants) will resist to the aridity of this area. The reserve is under an arid climate, erratic rains fall between November and March with the average of 238 mm per year (±88.27, n = 52). The temperature ranges from 5°C in January to near 40°C in July and August.

### Feces Sampling and Plant Inventory

A total of 60 feces samples were collected in three times during the peak of annual vegetation production (spring: 22–27 march, 2009), the middle (summer: 25 July–02 August, 2009), and the end of the dry season (autumn: 15–19 October, 2009) for analysis. The 20 feces samples collected each season were preserved dry in silica gel and the fecal samples older than one week were excluded in order to optimize the cost and benefit of the genetic analyses. Moreover, for each fecal sample, the sampling date and the geographical coordinates were recorded by a GPS receiver (Garmin *eTrex*).

Samples of all plant species encountered in the reserve in the three studied seasons were collected, identified to the lowest taxonomic level using identification keys [41]–[47] and the list of plant species occurring in the region.

### DNA Extraction from Feces

All extractions were performed in a room dedicated to nucleic acids extraction. Total DNA was extracted from about 10 mg of feces with the DNeasy Blood and Tissue Kit (QIAgen GmbH, Hilden, Germany), following the manufacturer’s instructions. The DNA extracts were recovered in a total volume of 250 µL. Mock extractions without samples were systematically performed to monitor possible contaminations.

### DNA Amplification

DNA amplifications were carried out in a final volume of 50 µL, using 4 µL of DNA extract diluted 100 times as template. The amplification mixture contained 1 U of AmpliTaq® Gold DNA Polymerase (Applied Biosystems, Foster City, CA), 10 mM Tris-HCl, 50 mM KCl, 2 mM of MgCl2, 0.2 mM of each dNTP, 0.1 µM of each primer, and 0.005 mg of bovine serum albumin (BSA, Roche Diagnostic, Basel, Switzerland). The mixture was denatured at 95°C for 10 min, followed by 45 cycles of 30 s at 95°C, and 30 s at 55°C; as the target sequences are usually shorter than 100 bp, the elongation step was removed to reduce the +A artifact [48], [49] that might decrease the efficiency of the first step of the sequencing process (blunt- end ligation). Samples were amplified using three primer pairs (Table 1). The first pair (*g* and *h*) corresponds to a universal approach, and targeted the P6 loop region of the *trn*L (UAA) intron [39]. In order to increase the resolution of the analysis, two other primer pairs were used. Both targeted the first internal transcribed spacer (ITS1) of nuclear ribosomal DNA, for Poaceae (ITS1-F and ITS1Poa-R) and for Asteraceae (ITS1-F and ITS1Ast-R). All primers were modified by the addition of specific tags on the 5′ end to allow the assignment of sequence reads to the relevant sample [4]. As a consequence, all the PCR products were tagged identically on both ends. These tags were composed of CC on the 5′ end followed by nine variable nucleotides that were specific to each sample. The nine variable nucleotides were designed using the oligoTag program (www.prabi.grenoble.fr/trac/OBITools) with at least three differences among the tags, without homopolymers longer than two, and avoiding a C on the 5′ end. All the PCR products from the different samples were first titrated using capillary electrophoresis (QIAxel, QIAgen GmbH, Hilden, Germany) and then mixed together, in equimolar concentration, before the sequencing.

**Table 1:** Primers used in the present study.

Name	Primer sequence (5′- 3′)	Reference
G	GGGCAATCCTGAGCCAA	Taberlet et al. (2007)
H	CCATTGAGTCTCTGCACCTATC	Taberlet et al. (2007)
ITS1-F	GATATCCGTTGCCGAGAGTC	This study
ITS1Poa-R	CCGAAGGCGTCAAGGAACAC	This study
ITS1Ast-R	CGGCACGGCATGTGCCAAGG	This study

### DNA Sequencing

The sequencing was carried out on the Illumina/Solexa Genome Analyzer IIx (Illumina, San Diego, California), using the Paired-End Cluster Generation Kit V4 and the Sequencing Kit V4 (Illumina, San Diego, California), and following manufacturer’s instructions. A total of 108 nucleotides were sequenced on each extremity of the DNA fragments.

### Sequence Analysis and Taxon Assignation

The sequence reads were analyzed using the OBITools (www.prabi.grenoble.fr/trac/OBITools). First, the direct and reverse reads corresponding to a single molecule were aligned and merged using the solexaPairEnd program, taking into account quality data during the alignment and the consensus computation. Then, primers and tags were identified using the ngsfilter program. Only sequences with perfect match on tags and a maximum of two errors on primers were taken into account. The amplified regions, excluding primers and tags, were kept for further analysis. Strictly identical sequences were clustered together using the obiuniq program, keeping the information about their distribution among samples. Sequences shorter than 10 bp, or containing nucleotides other than A, C, G and T, or with occurrence lower or equal to 10 were excluded using the obigrep program. Taxon assignation was achieved using the EcoTag program [36]. EcoTag relies on an exact global alignment algorithm [50] to find highly similar sequences in the reference database. This database was built by extracting the P6 loop of the *trn*L intron from EMBL nucleotide library using the ecoPCR program [51]. A unique taxon was assigned to each unique sequence. This unique taxon corresponds to the last common ancestor node in the NCBI taxonomic tree of all the taxids annotating the sequences of the reference database that matched against the query sequence. Automatically assigned taxonomic identification were then manually curated to further eliminate a few sequences that probably resulted either from PCR artifacts or that did not correspond to any plant P6 loop or ITS1 sequences present in the EMBL database (homology <0.9). Sequences with a total number of occurrence lower than 1/1000 for the P6 loop and ITS1 (Poaceae), or lower than 1/100 for ITS1 (Asteraceae) were also removed. Finally, the comparison between (i) the identification originating from the database built from EMBL and (ii) the list of plant species occurring within the geographic range of the gazelles allowed improving the final identification by excluding three taxa (*Cedrus sp*., *Arachis sp*., *Actinidia sp*.) observed at very low percentage, that are not present in the area, and that corresponded to obvious contaminations.

## Results

The 60 fecal pellet samples analyzed were successfully typed at the *trn*L locus and gave consistent results. For all these 60 samples, a total of 859933 sequence reads were obtained, with an average of 14332.22±8635.58 sequence reads per sample.

The three seasons DNA-based diet analyses of the Moroccan dorcas gazelle from the MT Reserve are summarized in Table S1. The main food composition on the studied three seasons consisted of 71 plant taxa, belonging to 57 genera and 29 families. Sixty-three percent of plants were identified at species level, 83% at genus level and 100% at their family level. The dicotyledons were dominant in all samples, with occurrence frequencies of 77.5% in spring, 85.4% in summer and 73.5% in autumn. Herbs prevailed (about 85.6%) in the diet of the Moroccan dorcas gazelle and were more frequently eaten in spring and autumn and relatively less in summer (Occurrence frequencies of 87.5 and 91.2 vs. 78%).

**Figure 2:**
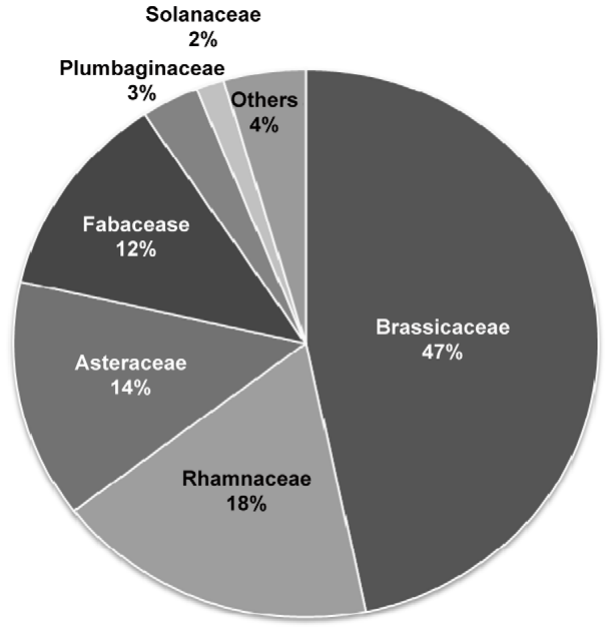
Proportions of the main plant families in Moroccan dorcas Gazelles diet from West central Morocco. Data are based on sequence variation of the P6 loop of the chloroplast *trn*L (UAA) intron using feces (collected in the M’Sabih Talaa Reserve) as source of DNA.

In spring, the diet is based on 40 taxa with only 25% of them (7 species: *Anagallis arvensis, Limonium thouini, Acacia gummifera, Erodium salzmannii, Lotus arenarius, Diplotaxis tenuisiliqua, Eruca sativa*, 1 taxon belonging to the genus *Diplotaxis* and 2 unidentified taxa of the family Brassicaceae) occurring at least in 50% of samples. In summer, gazelle’s food was made of 41 taxa with only 9 taxa present in the half of considered samples (4 species: *Glebionis coronaria, Cladanthus arabicus, Diplotaxis tenuisiliqua, Erodium salzmannii, Zizyphus lotus*,1 taxon of the genus *Alyssum* and 3 taxa belonging respectively to three families: Amaranthaceae, Asteraceae and Brassicaceae). In autumn, dorcas gazelle feed on 49 taxa of which only 18% occurring in at least 50% of samples (*Glebionis coronaria, Cladanthus arabicus, Acacia gummifera, Erodium salzmannii, Zizyphus lotus* and 4 taxa affiliated with four families: Amaranthaceae, Asteraceae, Brassicaceae and Malvaceae). The proportions of the main families occurring in the diet are illustrated in Figure 2. About 91% of the diet composition is made of plant taxa from four families: Brassicaceae (47%) followed by Rhamnaceae (18%), Asteraceae (14%) and Fabaceae (12%); the remaining proportion included Plumbaginaceae (3%), Solanaceae (2%) and 11 other families with less than 1% each (a total of 4%).

## Discussion

The present study constitutes a re-evaluation of the Moroccan dorcas gazelle diet in the MT Reserve after the first study carried out by Loggers [31] in the middle of 1980s using the microhistological examination of fecal material, which is among the most frequently used methods of determining the food habits in large herbivores. However, diets determined by microhistological fecal analysis are not as diverse as those determined by the other more recent methods since several minor forbs were not found in feces [9], [52], [53]. This may be explained partially by differential digestibility among plant species. In fact, it is conceivable that certain species of forbs are entirely digested, leaving no residue in the feces [54], [55]. In other instances plant fragments were present but were so transparent that cellular structure was not easily recognizable. Other divergences must be attributed to the proportion of the different plant groups in the diets which will be biased towards the most easily identified groups given that a substantial fraction of the stomach content is left unidentified by the microhistological diet analysis. Another bias is due to varying epiderm/mesophyll ratios between taxa [38]. Finally, the observer subjectivity in the microhistological identification processes may constitute another general problem [4].

In the present study, we evaluated the Moroccan dorcas gazelle’s diet using a quite new approach, the *trn*L approach (a DNA-based method). This technique is in most cases applicable to all herbivorous species eating angiosperms and gymnosperms [4]. It has been proved to be robust and reliable on the basis the very short length of the amplified region. The primers target highly conserved regions in angiosperms and gymnosperms, preventing strong bias due to primer mismatch in the efficiency of amplifications among species [39]. So, this DNA based technique gave by far a taxonomically more detailed picture of the diet composition than did the microhistological analysis. In this regard, as suggested by Valentini et al. [4] and in order to increase the resolution in taxa where sequences do not vary among the species (e.g., Asteraceae, Poaceae), we designed two new primers (see table 1). In spite of all these differences, there was an agreement between this DNA based method and microhistological analysis with respect to the importance of the main plant groups. Also, we can conclude that the large change detected in the Moroccan dorcas gazelle’s food selection cannot be attributed only to the bias due to the microhistological technique. This is suggested by the fact that only an average of 10 taxa were common between the two compared periods (1986–87 and 2009) and up to 34 taxa was newly identified in the diet while 13 were not encountered in the diet in 2009. These latters have probably become less abundant or totally disappeared from the reserve. The decrease in the plant specific richness in the reserve during the last two decades as indicated by the lower number of plant species in 2009 (130 vs. 170 species in the 1980s), could be attributed partially to the effect of successive drought periods, but also to the differences in phenology between the plant species which requires a more complete survey of the plant inventory throughout the whole year.

Overall, when comparing our results to those reported by Loggers [31] for the same population in the MT Reserve, 22 years ago (1986–1987), the diet composition revealed to be presently (2009) much more diversified (71 vs. 29 identified taxa) with 40–41 taxa in spring- summer and 49 taxa in autumn vs. respectively 23–22 and 26 in 1986–1987. The corresponding specific richnesses in the whole reserve were 130 and 171 species, respectively. Identified taxa differed markedly between the two periods (2009 and 1986–87) with numbers of common taxa of only 8, 10 and 11, for spring, summer and autumn, respectively. The main shared taxa are one grass species (*Stipa retorta*), three forb taxa (*Cladanthus arabicus, Plantago spp.* and *Medicago spp*.), and three shrub species (*Acacia gummifera, Atriplex halimus* and *Zizyphus lotus*).

The major factors influencing food selection in herbivores are mainly the energy, water and toxic contents of consumed plants (e.g., Pyke [56] for a review and Moskovits and Bjorndal [57], Lagarde et al. [58] and Mason et al. [59]. Belovsky [60] and Owen-Smith and Novellie [61] recognized that concentrations of specific nutrients in potential foods may be determinant in diet choice. Henley and Ward [34] found that fecal crude protein percentage in dorcas gazelle in the Negev desert, Israel, was significantly higher than that of the plant species which, according to these authors, implies that dorcas gazelle have a diet substantially richer in crude protein than the mean quality that is available in the forage. This difference is likely to be due to selection of plant parts that are higher in protein content than the mean quality for the sum of plant parts collected. This suggests that dorcas gazelle may diversify their diet composition in order to by-pass a diet becoming not compatible with its nutritional needs. In the present study, and relatively to the first determination of the diet in the same gazelle population in the 1980s [31], we found that the diet composition has become much more diversified irrespective of the discrepancy in taxonomic resolution between the methods used. Such a diversification might be related to a quantitative and qualitative increase of the food intake which constitutes the common answer of the majority of herbivores to a decrease of the nutritional value of their food as a result of the potential impact of climate change, especially drought and the increased CO_2_ concentration of the atmosphere [62]. Indeed, several studies showed a decrease in the nutritional quality of plants under an atmosphere enriched in CO_2_. This decline of the nutritional quality, especially in leaves, can reach 10–30% of the nitrogen causing an increase in the Carbon/Nitrogen ratio and consequently less nourishing leaves for herbivores [63]–[74]. This can be also perceived in the transformation of carbon hydrates becoming defense elements such as terpens and phenolic compounds [75], [76] and in condensed tannins which influence the digestibility [64], [77], [78]. Elevated CO_2_ should also cause a slight decrease in nitrogen-based defenses (e.g., alkaloids) and a slight increase in carbon-based defenses (e.g., tannins) in diverse plants species [62]. This would have certainly an effect on their selection by gazelles, in particular in period of drought where the food consumption would become more important because of the decrease of their nutritional quality.

Some plant species presenting a risk of toxicity for mammalian herbivores such as *Anagallis arvensis, Lotus arenarius* and *Chenopodium murale* [79]–[81] were detected in the diet of dorcas gazelle in the MT Reserve, suggesting that dorcas gazelle may even consume toxic plants which could be rich in some potentially required nutrients. This could be also interpreted as an anti-parasitic strategy. However, it has been reported that dorcas gazelle in the Negev desert can feed on the lily *Pancratium sickenbergeri* (Amarylidaceae) that is very toxic due to a high content of oxalate calcium; but the gazelle seems to eat only those parts of the leaves where calcium oxalate raphides are absent [82].

From the viewpoint of range management and conservation of this endangered remnant Moroccan dorcas gazelle population, it should be necessary to preserve the floristic diversity existing in the reserve. The shrub species, namely *Acacia gummifera*, *Atriplex halimus* and *Zizyphus lotus*, which are of a considerable nutritional importance during the dry season, should be particularly promoted in the reforestation programs. Moreover, as it has been suggested that the MT dorcas gazelle population should be split into two subpopulations in order to reduce its susceptibility to environmental stochasticity [83] the selection of the new potential site which would host the translocated subpopulation, should have similar diverse plant communities including most of the eaten species.

“Data deposited in the Dryad Repository: http://dx.doi.org/10.5061/dryad.6r42r366”.

## Supporting Information

Table S1 **Plant taxa identified in the Moroccan dorcas gazelle’s diet in the M’Sabih Talaa reserve, Morocco.** Data are based on sequence variation of the P6 loop of the chloroplast *trnL* (UAA) intron using feces as a source of DNA. %OF: occurrence frequency in %.
(XLS)

## References

[1] Duffy JE, Cardinale BJ, France KE, McIntyre PB, Thébault E (2007). The functional role of biodiversity in ecosystems: incorporating trophic complexity.. Ecol Lett.

[2] Bradley B, Stiller M, Doran-Sheehy D, Harris T, Chapman C (2007). Plant DNA sequences from feces: Potential means for assessing diets of wild primates.. Am J Primatol.

[3] Cristóbal-Azkarate J, Arroyo-Rodríguez V (2007). Diet and activity pattern of howler monkeys (*Alouatta palliata*) in Los Tuxtlas, Mexico: effects of habitat fragmentation and implications for conservation.. Am J Primatol.

[4] Valentini A, Miquel C, Nawaz N, Bellemain E, Coissac E (2009). New perspectives in diet analysis based on DNA barcoding and parallel pyrosequencing: the *trn*L approach.. Mol Ecol Resour.

[5] Gordon IJ (1995). Animal-based techniques for grazing ecology research.. Small Ruminant Res.

[6] Moreno-Black G (1978). The use of scat samples in primate diet analysis.. Primates.

[7] Van Wyk J (2000). Seasonal variation in stomach contents and diet composition in the large girdled lizard, *Cordylus giganteus* (Reptilia: Cordylidae) in the Highveld grasslands of the northeastern Free State, South Africa.. Afr Zool.

[8] Holechek JL, Vavra M, Pieper RD (1982). Botanical Composition Determination of Range Herbivore Diets: A Review.. J Range Manage.

[9] McInnis ML, Varva M, Krueger WC (1983). A comparison of four methods used to determine the diets of large herbivores.. J Range Manage.

[10] Duncan AJ, Mayers RW, Lamb CS, Young SA, Castillo I (1999). The use of naturally occurring and artificially applied n-alkanes as markers for estimation of short-term diet composition and intake in sheep.. J Agr Sci.

[11] Hutchings MR, Gordon IJ, Robertson E, Kyriazakis I, Jackson F (2000). Effects of parasitic status and level of feeding motivation on the diet selected by sheep grazing grass/clover swards.. J Agr Sci.

[12] Salt CA, Mayes RW, Elston DA (1992). Effects of season, grazing intensity and diet composition on the radiocaesium intake by sheep on re-seeded hill pasture.. J Appl Ecol.

[13] Bugalho MN, Milne JA, Racey PA (2001). The foraging ecology of red deer (*Cervus elaphus*) in a Mediterranean environment: is a larger body size advantageous?. J Zool.

[14] Hulbert IA, Iason GR, Mayes RW (2001). The flexibility of an intermediate feeder: dietary selection by mountain hares measured using n-alkane analysis.. Oecologia.

[15] Rao SJ, Iason GR, Hulbert IAR, Mayes RW, Racey PA (2003). Estimating diet composition for mountain hares in newly established native woodland: development and application of plant-wax faecal markers.. Can J Zoolog.

[16] Dove H, Mayes RW (1996). Plant wax components: A new approach to estimating intake and diet composition in herbivores.. J Nutr.

[17] Foley WJ, McIlwee A, Lawler I, Aragones L, Woolnough AP (1998). Ecological Applications of near Infrared Reflectance Spectroscopy: A Tool for Rapid, Cost-Effective Prediction of the Composition of Plant and Animal Tissues and Aspects of Animal Performance.. Oecologia.

[18] Kaneko H, Lawler IR (2006). Can Near Infrared Spectroscopy Be Used To Improve Assessment Of Marine Mammal Diets Via Fecal Analysis?. Mar Mammal Sci.

[19] Floyd R, Abebe E, Papert A, Blaxter M (2002). Molecular barcodes for soil nematode identification.. Mol Ecol.

[20] Hebert PDN, Cywinska A, Ball SL, de Waard JR (2003). Biological identification through DNA barcodes.. P R Soc London.

[21] Hoss M, Kohn M, Paabo S, Knauer F, Schroder W (1992). Excrement analysis by PCR.. Nature.

[22] Hebert PDN, Gregory TR (2005). The Promise of DNA Barcoding for Taxonomy.. Syst Biol.

[23] Moritz C, Cicero C (2004). DNA Barcoding: Promise and Pitfalls.. PLoS Biol.

[24] IUCN S (2010). *Gazella dorcas*. In: IUCN 2010.. IUCN Red List of Threatened Species.Version.

[25] Cuzin F (1996). Réparation actuelle et statut des grands mammifères sauvages du Maroc (Primates, Carnivores, Artiodactyles).. Mammalia.

[26] Cuzin F (2003). Les grands Mammifères du Maroc méridional (Haut Atlas, Anti Atlas, Sahara)..

[27] Alados CL (1987). A cladistic approach to the taxonomy of the dorcas gazelle.. Israel J Zool.

[28] Ait Baamrane MA, Znari M, Loggers CO, Naimi M, El Mercht S (2009). Conservation and Management of an isolated remnant population of Moroccan Dorcas Gazelles North West of the Atlas Mountains..

[29] Loggers CO (1992). Population characteristics of dorcas gazelles in Morocco.. Afr J Ecol.

[30] Marraha M (1996). Utilisation du line transect dans l’estimation de la densité et des caractéristiques de la population de gazelle dorcas (*Gazella dorcas* L.) dans la réserve de M’Sabih Talaa.. Annales de la Recherche Forestière, Rabat Morocco.

[31] Loggers CO (1991). Forage availability versus seasonal diets, as determined by fecal analysis, of dorcas gazelles in Morocco.. Mammalia.

[32] Freeland WJ, Janzen DH (1974). Strategies in herbivory by mammals: the role of plant secondary compounds.. Am Nat.

[33] Westoby M (1978). What are the biological bases of varied diets?. Am Nat.

[34] Henley S, Ward D (2006). An evaluation of diet quality in two desert ungulates exposed to hyper-arid conditions.. Afr J Range For Sci.

[35] Kowalczyk R, Taberlet P, Coissac E, Valentini A, Miquel C (2011). Influence of management practices on large herbivore diet-Case of European bison in Bialowieza Primeval Forest (Poland).. Forest Ecol Manag.

[36] Pegard A, Miquel C, Valentini A, Coissac E, Bouvier F (2009). Universal DNA- Based Methods for Assessing the Diet of Grazing Livestock and Wildlife from Feces.. J Agr Food Chem.

[37] Rayé G, Miquel C, Coissac E, Redjadj C, Loison A (2011). New insights on diet variability revealed by DNA barcoding and high-throughput pyrosequencing: chamois diet in autumn as a case study.. Ecol Res.

[38] Soininen E, Valentini A, Coissac E, Miquel C, Gielly L (2009). Analysing diet of small herbivores: the efficiency of DNA barcoding coupled with high-throughput pyrosequencing for deciphering the composition of complex plant mixtures.. Front Zool.

[39] Taberlet P, Coissac E, Pompanon F, Gielly L, Miquel C (2007). Power and limitations of the chloroplast *trn*L (UAA) intron for plant DNA barcoding.. Nucleic Acids Res.

[40] Valentini A, Pompanon F, Taberlet P (2009). DNA barcoding for ecologists.. Trends Ecol Evol.

[41] Fennane M, Ibn Tattou M, Ouyahya A, El Oualidi J (2007). Flore Pratique du Maroc.. Travaux de l’Institut Scientifique.

[42] Fennane M, Ibn Tattou M, Mathez J, Ouyahya A, El Oualidi J (1999). Flore Pratique du Maroc.. Travaux de l’Institut Scientifique.

[43] Nègre R (1961). Petite flore des régions arides du Maroc occidental..

[44] Nègre R (1962). Petite flore des régions arides du Maroc occidental..

[45] Quezel P, Santa S (1962). Nouvelle flore de l’Algérie et des régions désertiques méridionales.. CNRS Paris Tome.

[46] Quezel P, Santa S (1963). Nouvelle flore de l’Algérie et des régions désertiques méridionales.. CNRS Paris Tome.

[47] Valdés B, Rejdali M, Achhal El Kadmiri A, Jury SL (2002). Cheklist of vascular plants of N Morocco with identification keys..

[48] Brownstein MJ, Carpten JD, Smith JR (1996). Modulation of non-templated nucleotide addition by Taq DNA polymerase: primer modifications that facilitate genotyping.

[49] Magnuson V, Ally D, Nylund S, Karanjawala Z, Rayman J (1996). Substrate nucleotide-determined non-templated addition of adenine by Taq DNA polymerase: implications for PCR-based genotyping and cloning.. Biotechniques.

[50] Needleman SB, Wunsch CD (1970). A general method applicable to the search for search for similarities in the amino acid sequence of two proteins.. J Mol Biol.

[51] Ficetola G, Coissac E, Zundel S, Riaz T, Shehzad W (2010). An *in* silico approach for the evaluation of DNA barcodes.. BMC Genomics.

[52] Korfhage RC (1974). Summer food habits of elk in the Blue Mountains of northeastern Oregon based on fecal analysis..

[53] Vavra M, Rice RW, Hansen RM (1978). A comparison esophageal fistula and fecal material to determine steer diets.. J Range Manage.

[54] Johnson MK, Pearson HA (1981). Esophageal, Fecal and Exclosure Estimates of Cattle Diets on a Longleaf Pine-Bluestem Range.. J Range Manage.

[55] Slater J, Jones RJ (1971). Estimation of the diets selected by grazing animals from microscopic analysis of the faeces-a warning.. J Aust I Agr Sci.

[56] Pyke GH (1984). Optimal Foraging Theory: A Critical Review.. Annu Rev Ecol S.

[57] Moskovits DK, Bjorndal KA (1990). Diet and Food Preferences of the Tortoises *Geochelone carbonaria* and *G. denticulata* in Northwestern Brazil.. Herpetologica.

[58] Lagarde F, Bonnet X, Corbin J, Henen B, Nagy K (2003). Foraging behaviour and diet of an ectothermic herbivore: *Testudo horsfieldi*.. Ecography.

[59] Mason MC, Kerley GIH, Weatherby CA, Branch WR (1999). Leopard Tortoises (*Geochelone pardalis*) in Valley Bushveld, Eastern Cape, South Africa: specialist or generalist herbivores?. Chelonian Conserv Bi.

[60] Belovsky GE (1981). Food Plant Selection by a Generalist Herbivore: The Moose.. Ecology.

[61] Owen-Smith N, Novellie P (1982). What Should a Clever Ungulate Eat?. Am Nat.

[62] Coley PD (1998). Possible Effects of Climate Change on Plant/Herbivore Interactions in Moist Tropical Forests.. Climatic Change.

[63] Fajer ED, Bowers MD, Bazzaz FA (1989). The Effects of Enriched Carbon Dioxide Atmospheres on Plant–Insect Herbivore Interactions.. Science.

[64] Kinney KK, Lindroth RL, Jung SM, Nordheim EV (1997). Effects of CO_2_ and NO_3_
^−^availability on deciduous trees: phytochemistry and insect performance.. Ecology.

[65] Lincoln DE, Couvet D, Sionit N (1986). Response of an insect herbivore to host plants grown in carbon dioxide enriched atmospheres.. Oecologia.

[66] Lincoln DE, Fajer ED, Johnson RH (1993). Plant-insect herbivore interactions in elevated CO_2_ environments.. Trends Ecol Evol.

[67] Lincoln DE, Sionit N, Strain BR (1984). Growth and Feeding Response *Pseudoplusia includens* (Lepidoptera: Noctuidae) to Host Plants Grown in Controlled Carbon Dioxide Atmospheres.. Environ Entomol.

[68] Oberbauer SO, Sionit N, Hastings SJ, Oechel WC (1986). Effects of CO_2_ enrichment and nutrition on growth, photosynthesis, and nutrient concentration of Alaskan tundra plant species.. Can J Botany.

[69] Lindroth RL, Arteel GE, Kinney KK (1995). Responses of Three Saturniid Species to Paper Birch Grown Under Enriched CO_2_ Atmospheres.. Funct Ecol.

[70] Osbrink WLA, Trumble JT, Wagner RE (1987). Host Suitability of *Phaseolus lunata* for *Trichoplusia ni* (Lepidoptera: Noctuidae) in Controlled Carbon Dioxide Atmospheres.. Environ Entomol.

[71] Reekie EG, Bazzaz FA (1989). Competition and patterns of resource use among seedlings of five tropical trees grown at ambient and elevated CO_2_.. Oecologia.

[72] Roth SK, Lingroth RL (1994). Effects of CO_2_ mediated changes in paper birch and white pine chemistry on gypsy moth performance.. Oecologia.

[73] Williams EE, Garbutt K, Bazzaz FA, Vitousek PM (1986). The response of plants to elevated CO_2_ IV. Two deciduous forest tree communities.. Oecologia.

[74] Wong SC (1979). Elevated atmospheric partial pressure of CO_2_ and plant growth.. Oecologia.

[75] Ayres MP (1993). Plant defense, herbivory and climate change..

[76] Williams RS, Lincoln DE, Thomas RB (1994). Loblolly pine grown under elevated CO_2_ affects early instar pine sawfly performance.. Oecologia.

[77] Norby RJ, O’Neill EG, Luxmoore RJ (1986). Effects of Atmospheric CO_2_ Enrichment on the Growth and Mineral Nutrition of *Quercus alba* Seedlings in Nutrient-Poor Soil.. Plant Physiol.

[78] Roth SK, Lindroth RL, Montgomery ME (1994). Effects of foliar phenolics and ascorbic acid on performance of the gypsy moth (*Lymantria dispar*).. Biochem Syst Ecol.

[79] Forshaw D (2000). “Redgut” a cause of deaths in sheep grazing lucerne and other legumes.. Animal Health, Animal Disease Surveillance Newsletter, The Agriculture Protection Program, December.

[80] Halsey LA (1998). Nitrate in forage cause cattle deaths: a common weed and uncommon circumstances, Managing nutrition and forages to improve productivity and profitability..

[81] Knight AP, Walter RG (2002). Plant causing sudden death.. Knight, A.P., Walter, R.G (Eds.), Plant Poisoning of Animals in North America.

[82] Ward D, Spiegel M, Saltz D (1997). Gazelle Herbivory and Interpopulation Differences in Calcium Oxalate Content of Leaves of a Desert Lily.. J Chem Ecol.

[83] Cuzin F, Sehhar EA, Watcher T (2007). Etude pour l’élaboration de lignes directrices et d’un plan d’action stratégique pour la conservation des ongulés au Maroc..

